# Maintenance of Intestinal Homeostasis in Diarrhea-Predominant Irritable Bowel Syndrome by Electroacupuncture Through Submucosal Enteric Glial Cell-Derived S-Nitrosoglutathione

**DOI:** 10.3389/fphys.2022.917579

**Published:** 2022-08-22

**Authors:** Yujun Hou, Ying Zhao, Huiling Jiang, Kai Wang, Wei Zhang, Siyuan Zhou, Ying Li, Qianhua Zheng

**Affiliations:** ^1^ Acupuncture and Tuina School, Chengdu University of Traditional Chinese Medicine, China; ^2^ Department of Rehabilitation Medicine, West China Hospital, Sichuan University, China; ^3^ Department of Traditional Chinese Medicine, the People's Hospital of Shifang, Shifang, China

**Keywords:** irritable bowel syndrome, electroacupuncture, enteric glial cell, S-nitrosothiols, intestinal barrier, occludin, ZO-1

## Abstract

**Objective:** To determine whether electroacupuncture (EA) maintains intestinal homeostasis in diarrhea-predominant irritable bowel syndrome (IBS-D) rats by repairing intestinal barrier function through enteric glial cell (EGC)-derived S-nitrosoglutathione (GSNO).

**Methods:** Sprague–Dawley rats were randomly divided into a control group (*n* = 10) and an IBS-D group (*n* = 20). These rats received senna solution by gavage and chronic unpredictable mild stress for 14 days and were further divided into a model group (*n* = 10) and an EA group (*n* = 10). Rats in the EA group were electroacupunctured at ST25 (Tianshu), ST36 (Zusanli), and LR3 (Taichong) for 20 min every day for 14 days. The abdominal withdrawal reflex (AWR), the percentage of time spent in open arms (OT%) in the elevated plus maze test, and the diarrhea index (DI) were measured. Histopathological examination was performed to evaluate the pathological features of the colon after sacrificing the rats. Transmission electron microscopy was used to observe the EGC in the muscle and submucosal layers. Enzyme-linked immunosorbent assay was performed to detect GSNO expression in the colon. Double immunofluorescence labeling was used to detect the colocalized GFAP and GSNO expressions in the muscle and submucosal layers. Plasma FITC-dextran was used to measure intestinal permeability, whereas western blot was used to detect ZO-1 and occludin expressions in the colon.

**Results:** OT% and ZO-1 and occludin expressions were significantly lower than those of the control group, whereas AWR scores, DI, GSNO expression in the colon, colocalized GFAP and GSNO expressions in the submucosal layer, and intestinal permeability were significantly higher than those of the control group. Structural EGC abnormalities were observed in the model group. After EA treatment, OT% and ZO-1 and occludin expressions increased significantly, whereas AWR scores, DI, GSNO expression, colocalized GFAP and GSNO expressions in the submucosal layer, and intestinal permeability decreased significantly. The EGC structure was then restored to its normal state.

**Conclusion:** EA treatment downregulates the submucosal EGC–derived GSNO expressions, repairs the intestinal barrier by upregulating the ZO-1 and occludin expression, and improves IBS-D symptoms, including visceral hypersensitivity, anxiety, and diarrhea, suggesting a potential role for EGC-derived GSNO in the regulation of intestinal homeostasis in IBS-D rats.

## 1 Introduction

Irritable bowel syndrome (IBS) is a common functional gastrointestinal disorder (FGID) characterized by abdominal discomfort and altered bowel habits (Ford et al., 2020). The high prevalence and difficulty to completely cure IBS result in an increased medical and financial burden (Black and Ford, 2020). In addition, the risk of anxiety and depression is much higher in patients with IBS (Pinto-Sanchez et al., 2017). Diarrhea-predominant IBS (IBS-D) is the most common IBS subtype. Medical treatments, including antidiarrheals, are often used as first-line agents in patients with IBS-D. However, most guidelines suggest against continuous use (Chey et al., 2015) because they may increase the risk of constipation and ischemic colitis (Quigley et al., 2016; Fukudo et al., 2021). Therefore, alternative therapies must be identified for this disorder.

As a crucial part of traditional Chinese medicine, acupuncture is increasingly used in the treatment of FGID. Evidence has shown that acupuncture was significantly associated with relief of FGID symptoms (Wang et al., 2021) and it can alleviate emotional symptoms in FGID patients better than pharmacotherapy (Wang et al., 2022). A randomized controlled trial with a large sample size exhibited the efficacy of acupuncture in treating IBS with no obvious side effects (Pei et al., 2020). Moreover, there are an increasing number of systematic reviews and meta-analyses to support that acupuncture may be a promising therapy for IBS (Huang et al., 2021). Thus, acupuncture can be considered an alternative treatment for this disease. As the most commonly adopted acupoints, ST36 (Zusanli), ST25 (Tianshu), and LR3 (Taichong) seem to play a positive role in relieving IBS symptoms, including abdominal pain, diarrhea, and emotional disturbances (Chen et al., 2019; Song et al., 2020; Zhao et al., 2022).

Visceral hypersensitivity and intestinal barrier dysfunction are the main etiologies of IBS-D (Saha, 2014). Enteric glial cell (EGC) is a principal component of the enteric nervous system (ENS) (Boesmans et al., 2019). A close association is present between EGC and intestinal barrier, visceral hypersensitivity, and ENS plasticity (Neunlist et al., 2013). In addition, an obvious EGC hyperactivity was observed in the colon of IBS-D rats (Zhao et al., 2022). Acupuncture may play a protective role in the function of intestinal barrier in IBS-D, which is very likely to connect to EGC, the central cell for intestinal homeostasis maintenance (Cheadle et al., 2013).

EA can relieve IBS-D symptoms by inhibiting EGC hyperactivation (Zhao et al., 2022) and promoting intestinal barrier function in IBS-D rats by upregulating ZO-1 expression (Chen et al., 2019). Our previous study has showed that EA could promote EGC morphologic repair, could inhibit the hyperactivation of EGC, and resulted in a significant decrease in EGC-derived substance P content (Zhao et al., 2022). In addition, it is reported that EA ST36 protects intestinal barrier integrity, partly *via* the activation of EGC (Hu et al., 2015). However, precise signaling targets have not been identified. Thus, further studies are required on EGC activation.

EGC secretions are crucial for intestinal barrier function. S-nitrosoglutathione (GSNO) (Meir et al., 2016; Bonetti et al., 2018) is one of the most vital secreted factors of EGC, which was shown to have an effect on intestinal barrier function (Hagbom et al., 2020) by regulating the expressions of tight junction proteins (Flamant et al., 2011); affecting cytoskeletal components, including F-actin (Savidge et al., 2007); regulating the phosphorylation of myosin light chain (Li et al., 2016); and inhibiting nuclear factor kappa-B expression and regulating oxidative stress (Turan et al., 2018). However, the effects of GSNO on intestinal barrier dysfunction in IBS-D have not been studied in detail.

**FIGURE 1:**
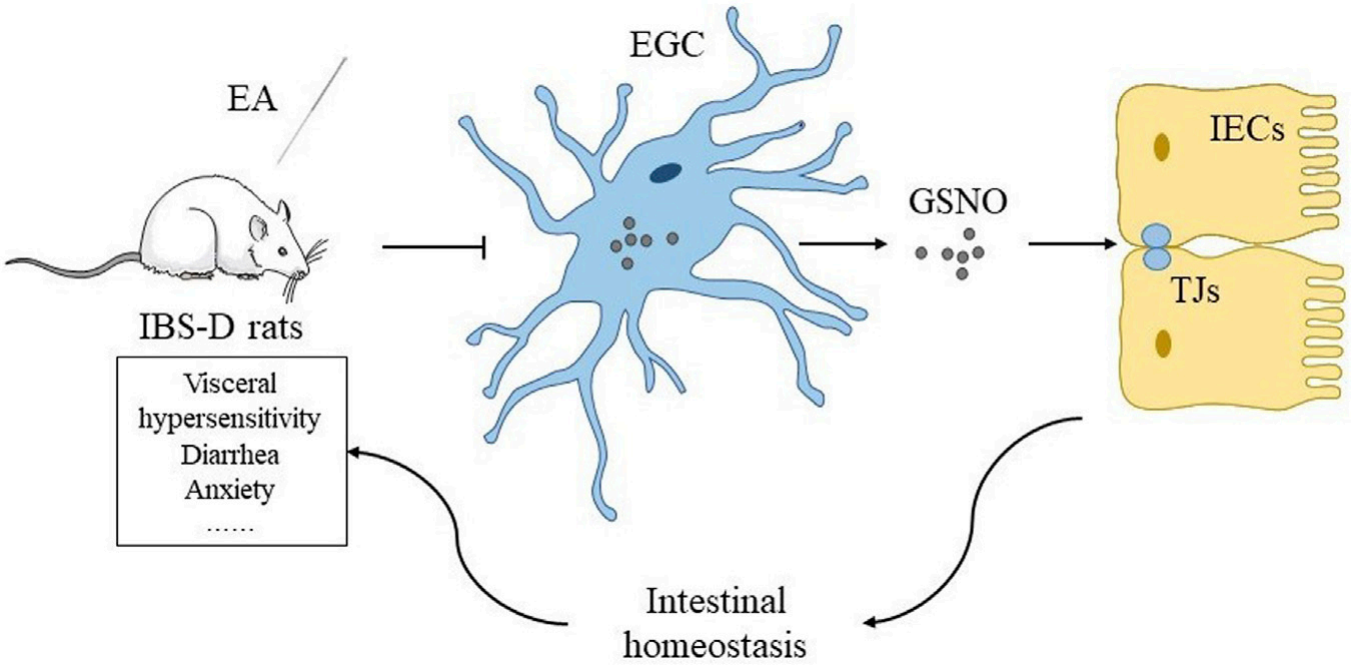
Hypothesis of the mechanism of EA maintaining intestinal homeostasis by regulating expressions of tight junction proteins through EGC-derived GSNO in IBS-D rats.

EA may downregulate EGC-derived GSNO expressions, repair the intestinal barrier by upregulating ZO-1 and occludin, restore intestinal homeostasis, and eventually relieve IBS-D symptoms such as visceral hypersensitivity, anxiety, and diarrhea (Figure 1).

## 2 Materials and Methods

### 2.1 Animals

The present study was conducted in 30 female Sprague–Dawley rats (aged 8 weeks, weighing 240 ± 10 g) obtained from the animal center of Chengdu Chinese Medicine University after institutional ethics clearance (reference No. 2019-06). The rats were housed with constant circadian rhythm, humidity, and temperature (20–25°C). Adequate water and food were provided.

### 2.2 Establishment of Irritable Bowel Syndrome-D Model

After 7 days of adaptation, rats were randomly divided into a control group (*n* = 10) and a chronic unpredictable mild stress (CUMS) group (*n* = 20). The IBS-D group rats were administered 0.3 g/ml of senna solution by gavage at 10 ml/kg and CUMS for 14 days. CUMS was administered by applying restraint for 2 h, tail clamping for 20 min, isolating for 24 h, placing in a crowded house for 24 h (more than six rats in one cage), applying warm water (45°C), swimming for 5–10 min, applying foot shock for 15 min, and oscillating swing for 1 h. These methods were randomly selected every day, and each method was administered twice and discontinuously for 14 days. After that, abdominal withdrawal reflex (AWR) scores were used to measure visceral hypersensitivity. The rats in the IBS-D group were further divided into a model group (*n* = 10) and an EA group (*n* = 10). The control group rats were provided distilled water by gavage at 10 ml/kg everyday for 14 days.

### 2.3 Electroacupuncture Treatment

ST36 (Zusanli), ST25 (Tianshu), and LR3 (Taichong), which are commonly used acupoints in IBS-D treatment, were selected in this study. ST36 is located at the posterior and lateral side of the knee joint, 5 mm under the fibula head. ST25 is located 5 mm lateral to the middle side of the umbilicus. LR3 is located between the first and second metatarsal bones on the foot dorsum. Rats were restrained by devices during treatment. Stainless steel acupuncture needles (Huatuo, Suzhou Medical Supplies Co., Ltd., Φ0.13 × 13 mm) were inserted into acupoints at a depth of 1–2 mm after disinfection, and an electroacupuncture (EA) apparatus (HANS-200A, Nanjing, China) was connected. The sparse and dense waves were set to an intensity of 1.5 mA and a frequency of 2 Hz/15 Hz. The treatment lasted for 20 min. The control and model groups were restrained using the same devices for 20 min everyday during treatment.

### 2.4 Model Evaluation

#### 2.4.1 Abdominal Withdrawal Reflex Score

AWR scores were used to evaluate visceral sensitivity. As described by Al-Chaer et al. (2000), rats were fasted for 24 h before the assessment. After anesthesia, the rats were put into an acrylic box. A balloon attached to a catheter was slowly inserted into the rat anus for 4.5 cm. After the rats recovered from anesthesia, the balloon was inflated at a uniform speed to reach 20, 40, 60, and 80 mmHg of pressure. Pressure was maintained for 30 s, and the AWR scores were recorded by a blinded researcher. The following five AWR scores (AWR 0–AWR 4) were used to assess the degree of visceral hypersensitivity during colorectal dilatation (CRD): AWR 0, no obvious behavior changes; AWR 1, unstable emotion and occasional head swing; AWR 2, slight abdominal muscle contraction; AWR 3, strong abdominal muscle contraction and abdomen raised; and AWR 4, body arching. Experiments were performed at least in triplicate, and the results were averaged. AWR scores were proportional to the visceral hypersensitivity level in rats.

#### 2.4.2 Elevated Plus Maze Test

The elevated plus maze test (EPMT) is used to measure anxiety-like behavior in rodents. In general, the amount of time spent in the open arms is compared to the amount of time spent in the open arms and closed arms as a measure of anxiety (Kraeuter et al., 2019). Rats were placed at the end of one open arm of the EPMT apparatus. After recording the activity of rats for 300 s, the times at which the rats entered the open arms and closed arms were recorded. The open arms time percentage (OT%) was calculated as follows: OT% = (open arms time/open arms time + closed arms time) × 100%. OT% was inversely proportional to the anxiety level of the rats (Zhang et al., 2017).

#### 2.4.3 Diarrhea Index

Diarrhea index (DI) was used to evaluate the severity of diarrhea. The number of loose stools, total number of stools, and area of stains were observed. Diarrhea index was calculated as follows:

Diarrhea index=loose stool rate×loose stool level

Loose stool rate=the number of loose stools/the total number of stools

The loose stool level was classified according to the loose stool diameter as follows: grade 1, <1 cm; grade 2, 1–2 cm; grade 3, 2–3 cm; and grade 4, >3 cm. DI was proportional to the severity of diarrhea in rats (Zhu et al., 2022).

### 2.5 Sample Collection

After EA treatment, rats were intraperitoneally injected with pentobarbital sodium (40 mg/kg). Distal colon (3 cm to the anus) and abdominal aorta blood were rapidly collected after anesthesia. The intestinal contents were washed with 0.9% normal saline (NaCl and distilled water). The cleaned colon was quickly placed in 4% paraformaldehyde (paraformaldehyde and 0.1 mol/L phosphate-buffered saline (PBS)) at room temperature for immunofluorescence detection, 10% neutral formaldehyde (sodium dihydrogen phosphate and sodium dihydrogen phosphate and formaldehyde solution) for histological assessment, 3% glutaraldehyde (Saint-Bio, China) for transmission electron microscopy detection, and liquid nitrogen for western blot (WB) detection. Plasma was obtained by centrifugation.

### 2.6 Intestinal Permeability Detection

Intestinal permeability was measured using the plasma FITC-dextran concentrations (Van Sebille et al., 2017). Rats were administered 20 mg/ml of FITC-dextran by gavage at 40 mg/kg 4 hours before sacrificed, and the abdominal aorta blood was collected and centrifuged at 3,000 rpm for 10 min, after which the resulting plasma was collected. Concentrations of plasma FITC-dextran were proportional to the intestinal permeability of the rats.

### 2.7 Histological Assessment

The colon sample (3 cm to the anus) was placed in 10% neutral formaldehyde, dehydrated with 100% alcohol in an automatic dehydrator, embedded in paraffin, sectioned, and stained with hematoxylin and eosin. The sample was observed under a light microscope (BA210 Digital, Motic, China)*.* The tissues were visualized under 40× objective lenses, images were collected from the selected areas at 400 times magnification, and the inflammatory grade was evaluated according to the pathological colon tissue (Table 1).

**TABLE 1:** Evaluation of colonic pathological tissue inflammation grade.

Systematics	Performance
− (0 mark)	No inflammation, no neutrophil infiltration in the propria, no edema in the interstitial
+ (1 mark)	Mild, with a small amount of neutrophil infiltration in the lamina propria, mild or no interstitial edema
++ (2 marks)	Moderate, moderate granulocyte infiltration in the lamina propria, moderate interstitial edema
+++ (3 marks)	Severe, moderate to large neutrophil diffuse infiltration in the lamina propria, severe interstitial edema

### 2.8 Enzyme-Linked Immunosorbent Assay

The colon tissues were collected and homogenized in PBS after centrifugation (5,000 × g, 10 min), and the supernatant was collected for analysis. The GSNO content in colon was determined by the enzyme-linked immunosorbent assay (ELISA) following the manufacturer’s instructions in the ELISA kit (ZCI BIO, China). The absorbance of each well (OD values) was measured at 450 nm, a standard curve was calculated by linear regression analysis, and the OD values measured for the samples were substituted into the linear regression equation of the standard curve to calculate the sample concentrations.

### 2.9 Western Blot

ZO-1 and occludin expressions were measured in the colon using WB. The total protein concentration was measured using the BCA protein quantification kit (Beyotime, China). Samples were separated by electrophoresis, transferred to a polyvinylidene difluoride membrane (Sigma-Aldrich, United States), and placed in 5% skimmed milk. The membrane was incubated with primary antibodies [anti-occludin antibody, 1:1000 dilution, rabbit clonal antibody (Affinity, United States); anti-ZO-1 antibody, 1:2000 dilution, rabbit clonal antibody (Abcam, United Kingdom); anti-β-actin antibody, 1:100000 dilution, rabbit clonal antibody (Abclonal, China)]. The membrane was incubated with a secondary antibody [biotinylated goat anti-rabbit IgG (H + L) 1:5000 dilution (Affinity, United States)] after washing. Relative occludin and ZO-1 expressions were obtained by normalization against actin.

### 2.10 Immunofluorescence

Double immunofluorescence labeling was used to determine the colocalization of GFAP and GSNO in the colon. After placement in 4% paraformaldehyde, the samples were sliced, immersed in 0.01 M citrate buffer (pH 6.0), heated until boiling, washed with PBS, and blocked with 10% serum (Zhejiang Tianhang Biotechnology, China) for 30 min. Primary antibodies [anti-GFAP, rabbit monoclonal antibody, 1:100 (Bioss, China); anti-GSONR, mouse monoclonal antibody, 1:100 (Proteintech, United States)] were added to the samples. After the samples were washed with PBS, a secondary antibody [FITC-labeled goat anti-rabbit IgG (ServiceBio, China); Cy3-labeled goat anti-mouse IgG (ServiceBio, China)] and 4′,6-diamidino-2-phenylindole were added and the samples were incubated at room temperature for 10 min, washed with PBS, sealed with fluorescence decay-resistant sealing tablets (AR1109, Boster, China), and examined under a fluorescence scanning microscope camera system (P250 Flash, Tangier, China).

### 2.11 Transmission Electron Microscopy

After the samples were prefixed with 3% glutaraldehyde, they were postfixed in 1% osmium tetroxide, dehydrated in series acetone, infiltrated in Epox 812, and embedded. Semithin sections were stained with methylene blue, whereas ultrathin sections were cut with a diamond knife and stained with uranyl acetate and lead citrate. Sections were examined under a transmission electron microscope (TEM) (JEM-1400Flash, Japan).

### 2.12 Statistical Analysis

SPSS 21.0 was used for statistical analysis. After normal test and homogeneity analysis of variance (ANOVA), one-way ANOVA, followed by a post-hoc least significant difference, was used. Mann–Whitney U test was used in cases where there were no variances among groups. Spearman correlation analysis was used to evaluate the correlation between the level of EGC-derived GSNO and intestinal barrier function. *P* ˂ 0.05 was considered statistically significant.

## 3 Results

### 3.1 Electroacupuncture Effect on Irritable Bowel Syndrome-D Symptoms

**FIGURE 2:**
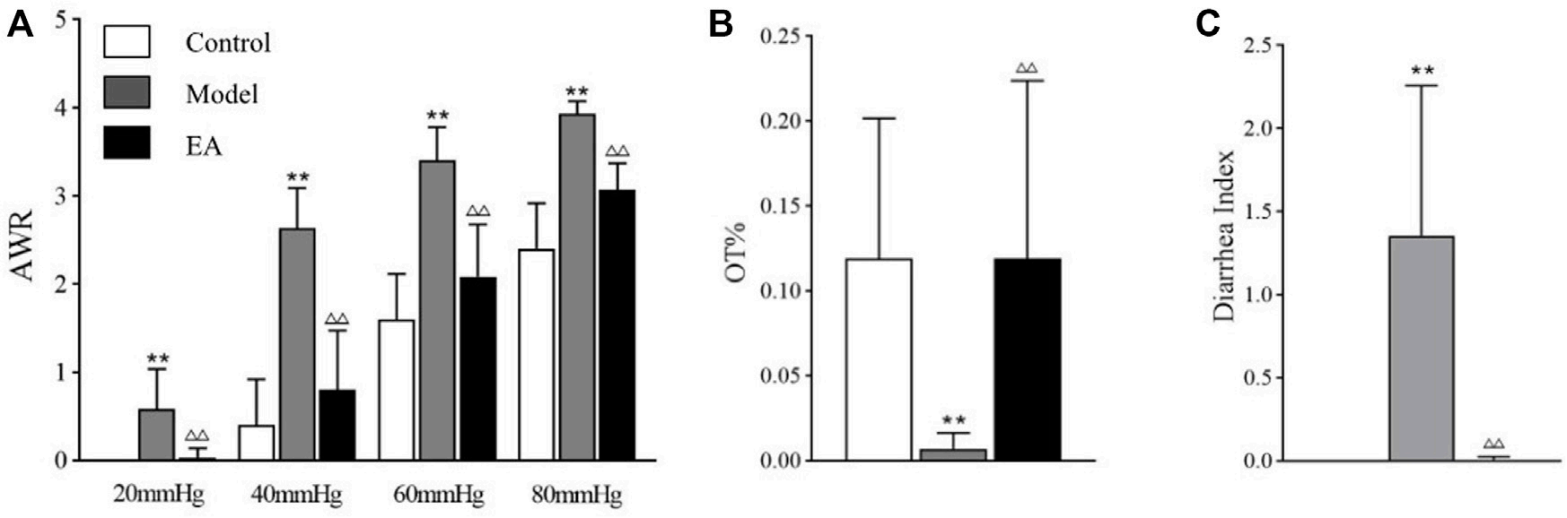
EA effect on IBS-D symptoms. Visceral hypersensitivity, anxiety-like behaviors, and the severity of diarrhea were measured using AWR scores **(A)**, OT% **(B)**, and DI **(C)**. one-way ANOVA indicated that all indexes were significantly higher in the model group (*p* < 0.01) and lower in the EA group. Bar: mean ± SE; *n* = 10; ^**^ *p* < 0.01, versus the control group; ^△△^ *p* < 0.01, versus the model group.

Visceral hypersensitivity was measured using AWR scores. Compared to the control group, AWR scores were significantly higher with CRD values of 20, 40, 60, and 80 mmHg in the model group (*p* < 0.01). After EA treatment, AWR scores exhibited a significant decrease at each pressure gradient (*p* < 0.01) (Figure 2A). OT%, the percentage of time spent in the open arms, is a crucial index to evaluate anxiety-like behaviors. OT% decreased significantly in the model group (*p* < 0.01), whereas OT% increased significantly after EA treatment (*p* < 0.01) (Figure 2B). After the stools were collected and observed, the severity of diarrhea was evaluated by DI. DI significantly increased in the model group (*p* < 0.01), whereas it significantly decreased after EA treatment (*p* < 0.01) (Figure 2C).

### 3.2 Histological Assessment

**FIGURE 3:**
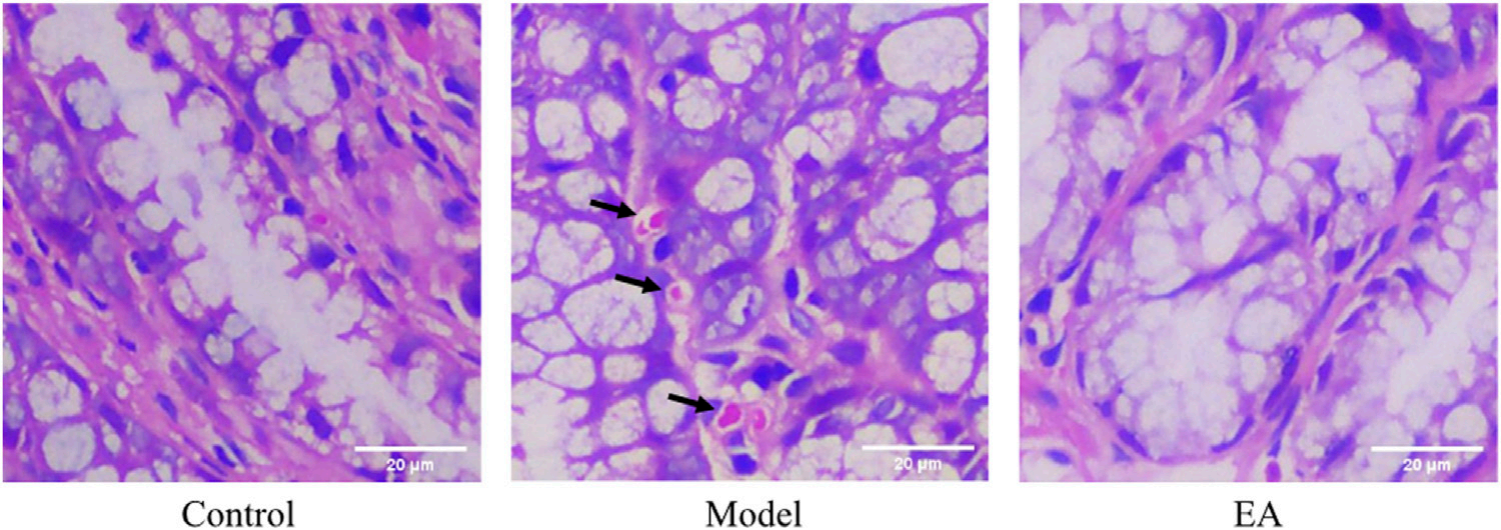
Histological assessment of each group. No obvious pathological changes were observed in the control group and EA group, whereas occasional scattered lymphocytes (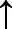) were observed in the model group. Images were taken under a light microscope (×400). Scale bar = 20 μm, *n* = 6.

Histological assessment exhibited that no obvious pathological changes were observed in the control group (Figure 3). Occasional scattered lymphocytes and no necrosis or other lesions were observed in the loose connective tissue of the model group (Figure 3). The tissue was complete and normal in the EA group (Figure 3). The mucosal layer, submucosal layer, muscle layer, and outer membrane structure of colon tissue were normal for all groups, and no obvious inflammation was observed. The inflammatory score of all groups was zero.

### 3.3 Electroacupuncture Effects on Enteric Glial Cell

#### 3.3.1 Electroacupuncture Effects on Enteric Glial Cell in the Muscle Layer

**FIGURE 4:**
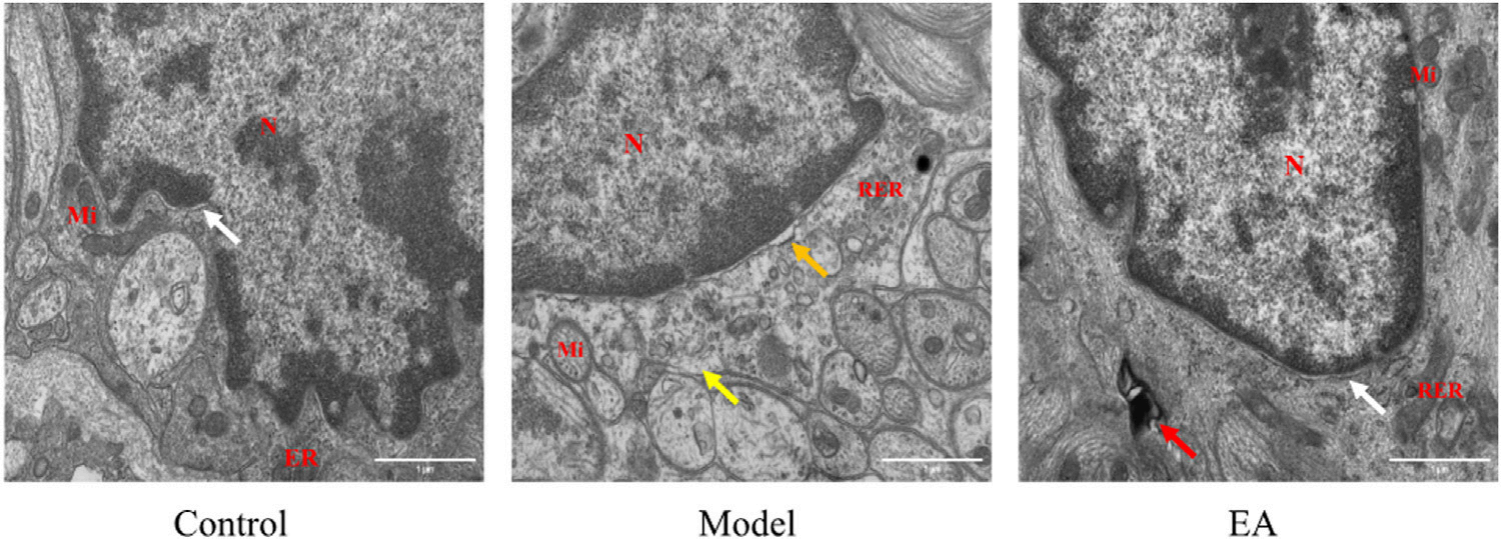
Morphology of EGC in the muscle layer under TEM (×30,000). N: nucleus, Mi: mitochondrion, RER: rough endoplasmic reticulum. In the muscle layer, a widened perinuclear space (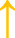) and ribosome loss (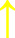) were observed in the model group, mild autophagy (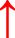) was observed in the EA group, and a normal perinuclear space (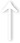) was observed both in the control and EA groups. Scale bar = 1 μm, *n* = 3.

The EGC morphology and structure in the muscle layer of the control group were normal (Figure 4 **)**. Loss of ribosome will affect protein synthesis and further cause cellular dysfunction. The model group exhibited loss of ribosome and widened perinuclear space (Figure 4). Mild autophagy was observed in the EA group (Figure 4).

#### 3.3.2 Electroacupuncture Effects on Enteric Glial Cell in the Submucosal Layer

**FIGURE 5:**
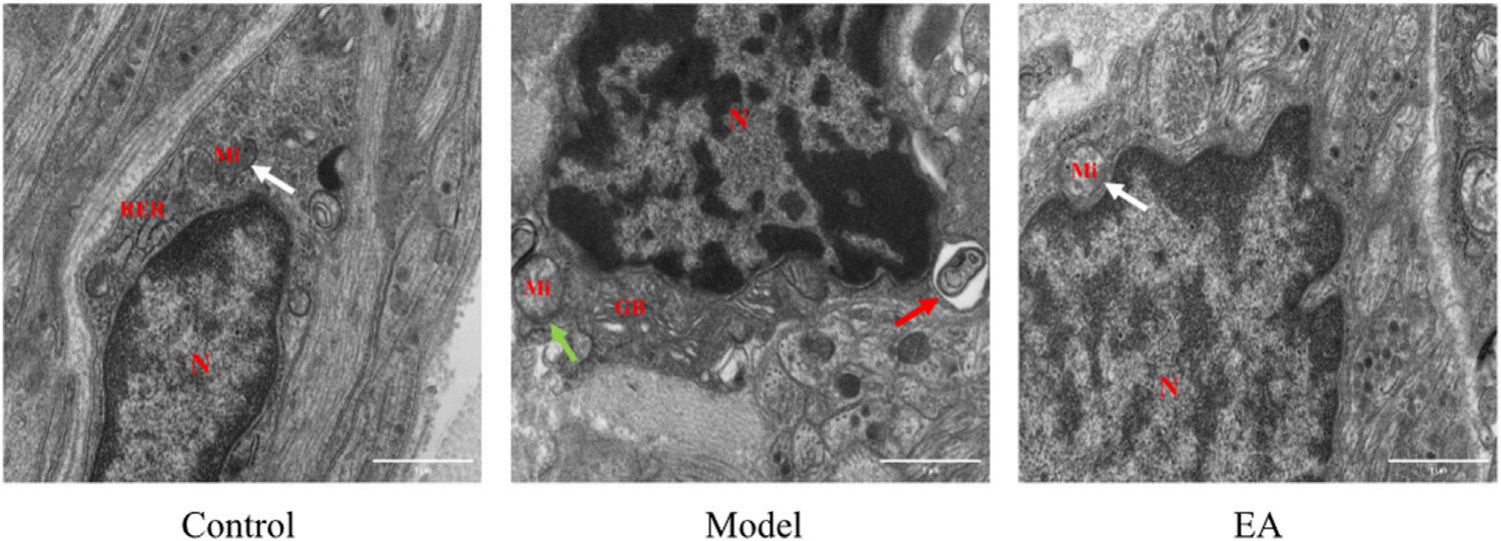
Morphology of EGC in the submucosal layer under TEM (×30,000). N: nucleus, Mi: mitochondrion, RER: rough endoplasmic reticulum, GB: Golgi body. In the submucosal layer, autophagy (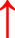) and mitochondrial swelling (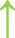) were observed in the model group, and normal mitochondrion (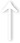) was observed in the control and EA groups. Scale bar = 1 μm, *n* = 3.

Normal EGC morphology and structure were observed in the submucosal layer of the control group (Figure 5) and EA group (Figure 5). The model group exhibited autophagy and mitochondrial swelling.

### 3.4 Electroacupuncture Effects on GFAP and S-nitrosoglutathione Expressions

#### 3.4.1 S-nitrosoglutathione Expression in the Colon

**FIGURE 6:**
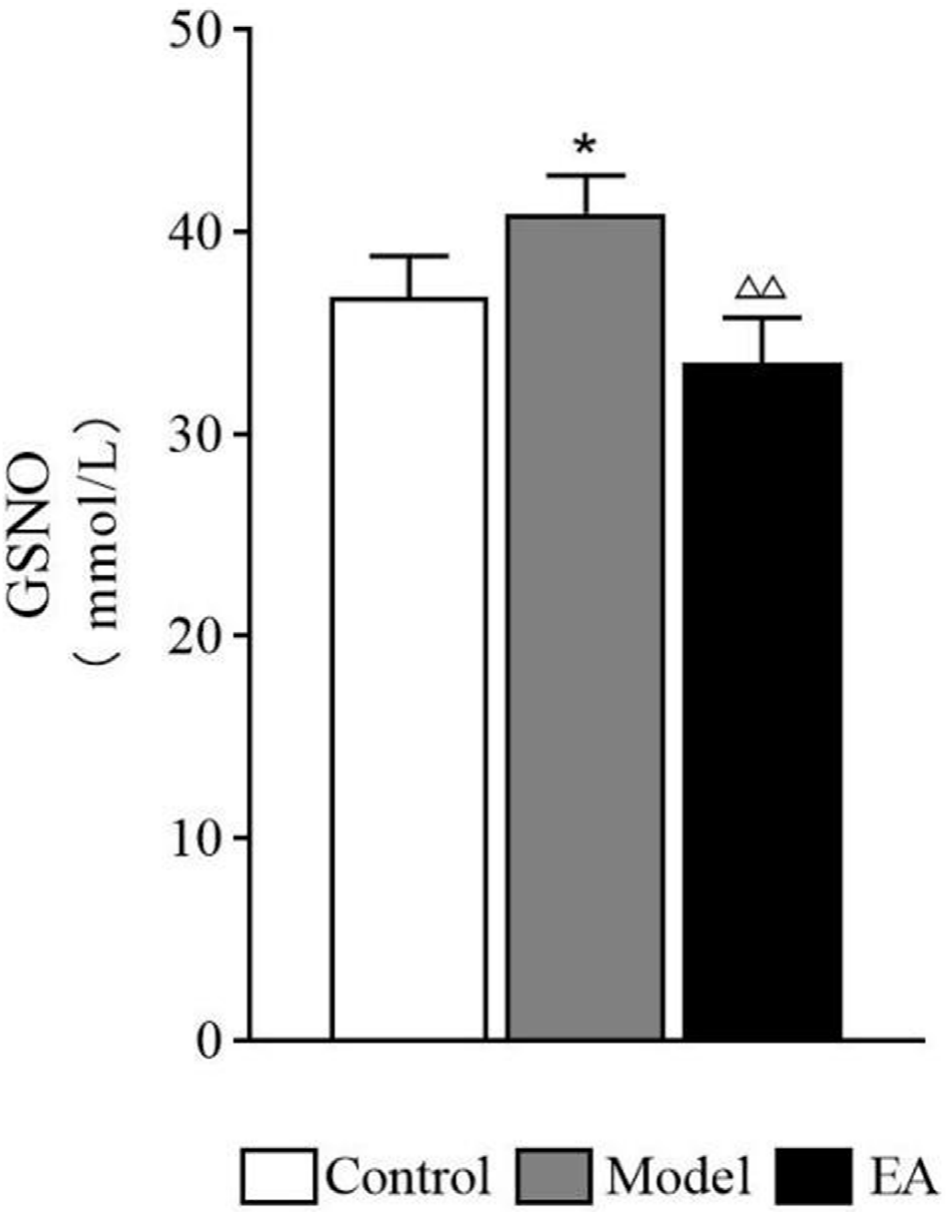
Expression of GSNO in the colon. One-way ANOVA indicated that the level of GSNO shows a significant increase after modeling and a significant decrease after EA treatment. Bar: mean ± SE, *n* = 4, ^*^ *p* < 0.05, versus the control group, ^△△^ *p* < 0.01, versus the model group.

The GSNO expression in the model group was significantly higher than that in the control group (*p* < 0.05). After EA treatment, a significant decrease in GSNO expression was observed in the EA group compared with the model group (*p* < 0.01) (Figure 6).

#### 3.4.2 GFAP and S-nitrosoglutathione Colocalization in the Muscle Layer

**FIGURE 7:**
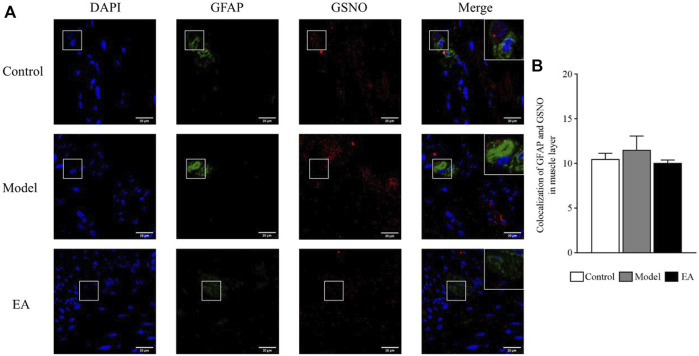
Colocalized GFAP and GSNO expressions in the muscle layer. GFAP and GSNO fluorescence in different groups **(A)**; green indicates GFAP, red indicates GSNO, and blue indicates DAPI-stained nucleus. One-way ANOVA indicated that the colocalization of GFAP and GSNO was not statistically different in different groups **(B)**. Scale bar = 20 μm, bar: mean ± SE, *n* = 4.

Double-label immunofluorescence assay was performed to determine the expression and location of EGC-derived GSNO (Figure 7A). The colocalized GFAP and GSNO expressions in the muscle layer was statistically nonsignificant between the groups (*p* > 0.05) (Figure 7B).

#### 3.4.3 GFAP and S-nitrosoglutathione Colocalization in the Submucosal Layer

**FIGURE 8:**
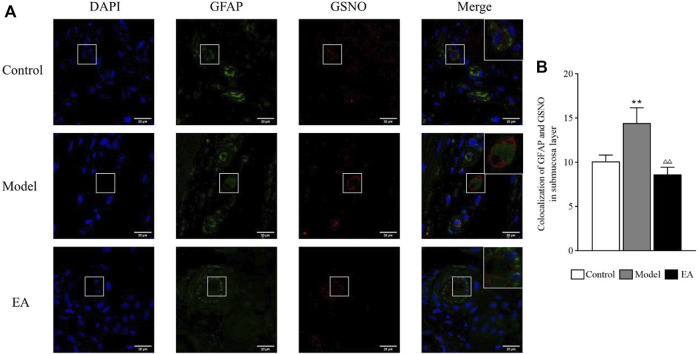
Colocalized GFAP and GSNO expressions in the submucosal layer. GFAP and GSNO fluorescence in different groups **(A)**; green indicates GFAP, red indicates GSNO, and blue indicates DAPI-stained nucleus. One-way ANOVA indicated that the colocalization of GFAP and GSNO was significantly higher in the model group and significantly lower after EA treatment **(B)**. scale bar = 20 μm, bar: mean ± SE, *n* = 4, ^**^ *p* < 0.01, versus the control group; ^△△^ *p* < 0.01, versus the model group.

A significant increase in colocalized GFAP and GSNO expressions was exhibited in the submucosal layer in the model group (*p* < 0.01). A significant decrease in their colocalization was observed postoperatively in the EA group (*p* < 0.01) (Figure 8).

### 3.5 Electroacupuncture Effect on Intestinal Barrier Function

#### 3.5.1 Occludin and ZO-1 Expressions in the Colon

**FIGURE 9:**
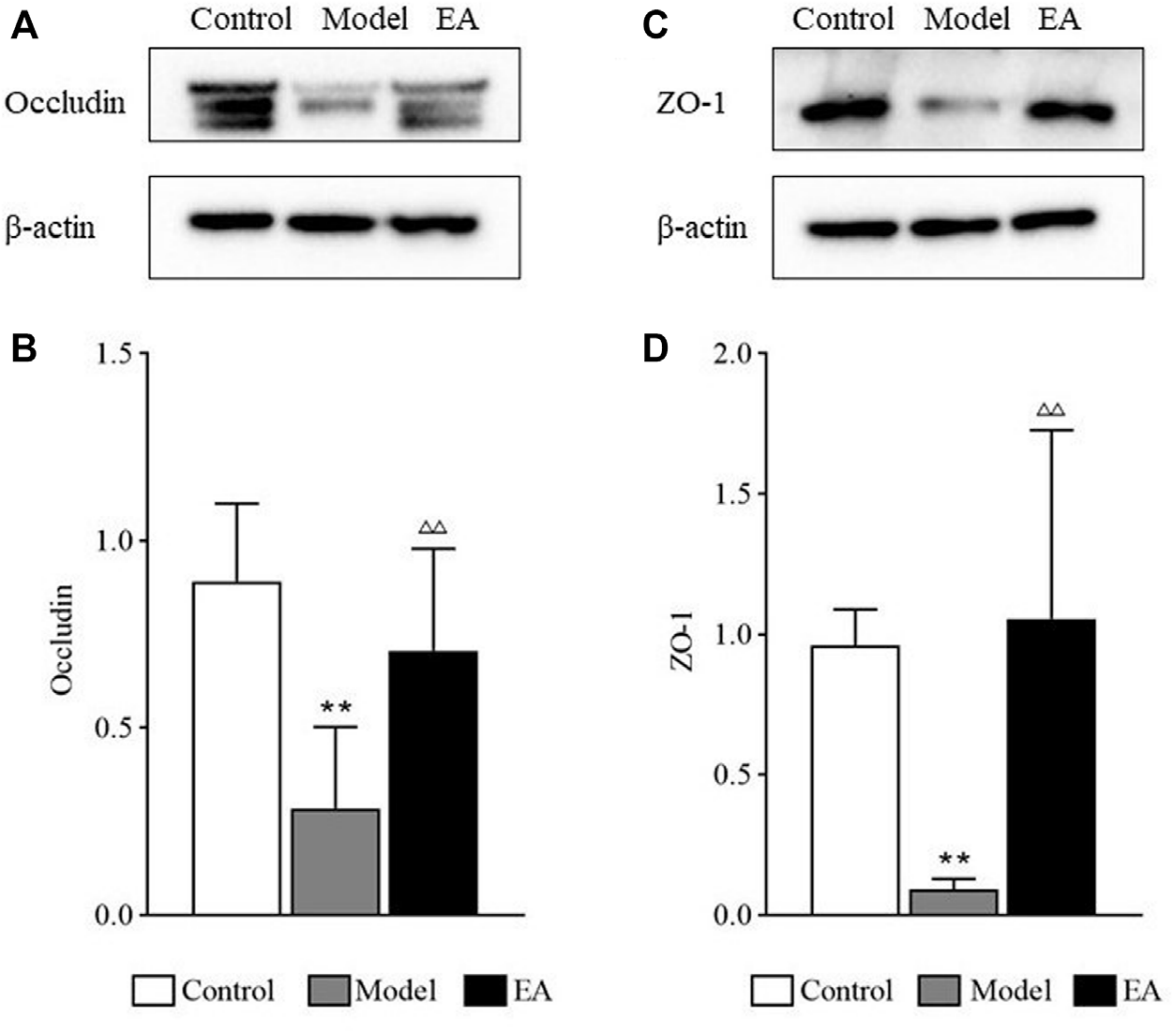
Tight junction proteins expressions. Occludin **(A)** and ZO-1 **(C)** expressions were measured by western blot. One-way ANOVA indicated that the expressions of occludin **(B)** and ZO-1 **(D)** were significantly lower after modeling and significantly higher after EA treatment. Bar: mean ± SE, *n* = 10, ^**^ *p* < 0.01, versus the control group; ^△△^ *p* < 0.01, versus the model group.

The occludin and ZO-1 expressions in the colon of the model group were significantly lower than those in the control group (*p* < 0.01). The expressions of these two tight junction proteins in the colon of the EA group were significantly higher than those in the model group (*p* < 0.01) (Figure 9).

#### 3.5.2 Concentrations of Plasma FITC-Dextran

**FIGURE 10:**
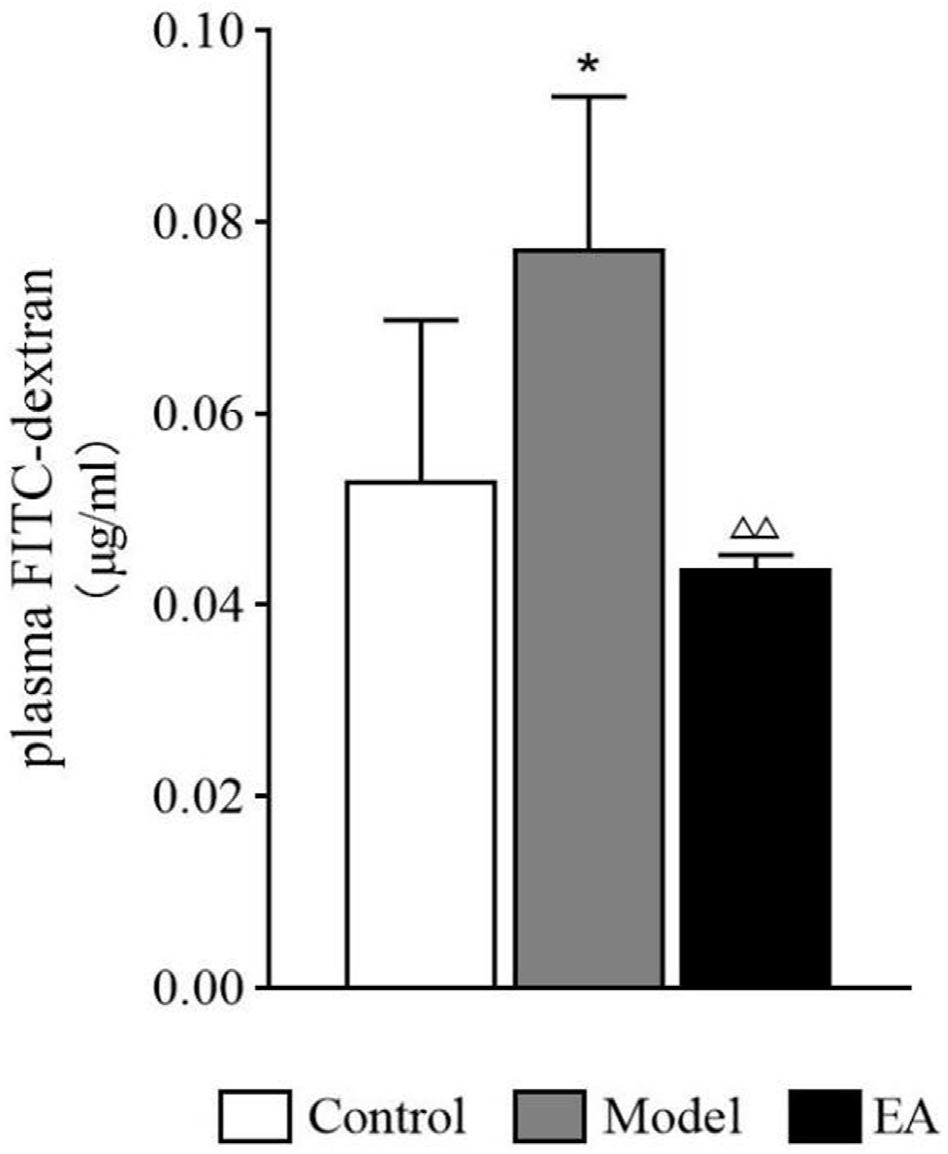
Concentrations of plasma FITC-dextran. One-way ANOVA indicated that the concentrations of plasma FITC-dextran showed a significant increase in the model group and a significant decrease in the EA group. Bar: mean ± SE, *n* = 4, *p < 0.05, versus the control group; ^△△^ *p* < 0.01, versus the model group.

After the rats were perfused and sacrificed, blood samples were obtained from the abdominal aorta. Intestinal permeability was measured using the concentrations of plasma FITC-dextran, which exhibited a significant increase in the model group (*p* < 0.05) and a significant increase after EA treatment (*p* < 0.01) (Figure 10).

### 3.6 Correlation Analysis Between the Level of Enteric Glial Cell-Derived S-nitrosoglutathione and Intestinal Barrier Function

#### 3.6.1 Muscle Layer

**FIGURE 11:**
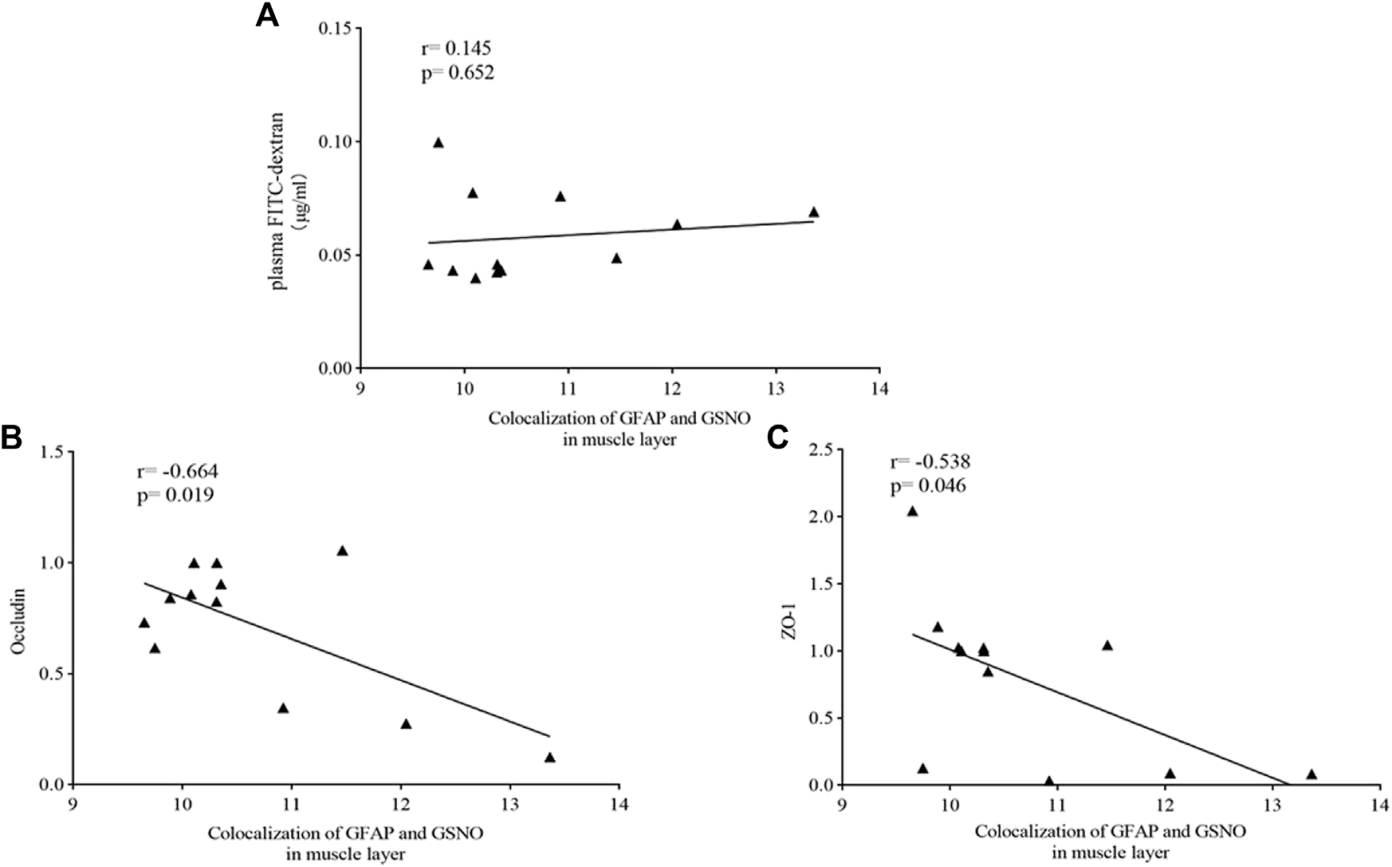
Correlation analysis between the level of EGC-derived GSNO in the muscle layer and intestinal barrier function. Spearman test showed that no significant correlations were observed between the expression of EGC-derived GSNO and of plasma FITC-dextran concentrations in the muscle layer **(A)**. an inverse correlation was observed between the level of EGC-derived GSNO in the muscle layer and the expressions of occludin **(B)** and ZO-1 **(C)**.

Spearman correlation analysis was used to evaluate the correlation between the level of EGC-derived GSNO and intestinal barrier function in the muscle layer. No significant correlations were observed between the expression of EGC-derived GSNO in the muscle layer and concentrations of plasma FITC-dextran (Figure 11A). However, a trend of inverse correlation was observed between the level of EGC-derived GSNO in the muscle layer and the expressions of occludin (r = −0.664, *p* = 0.019) (Figure 11B) and ZO-1 (r = −0.538, *p* = 0.046) (Figure 11C).

#### 3.6.2 Submucosal Layer

**FIGURE 12:**
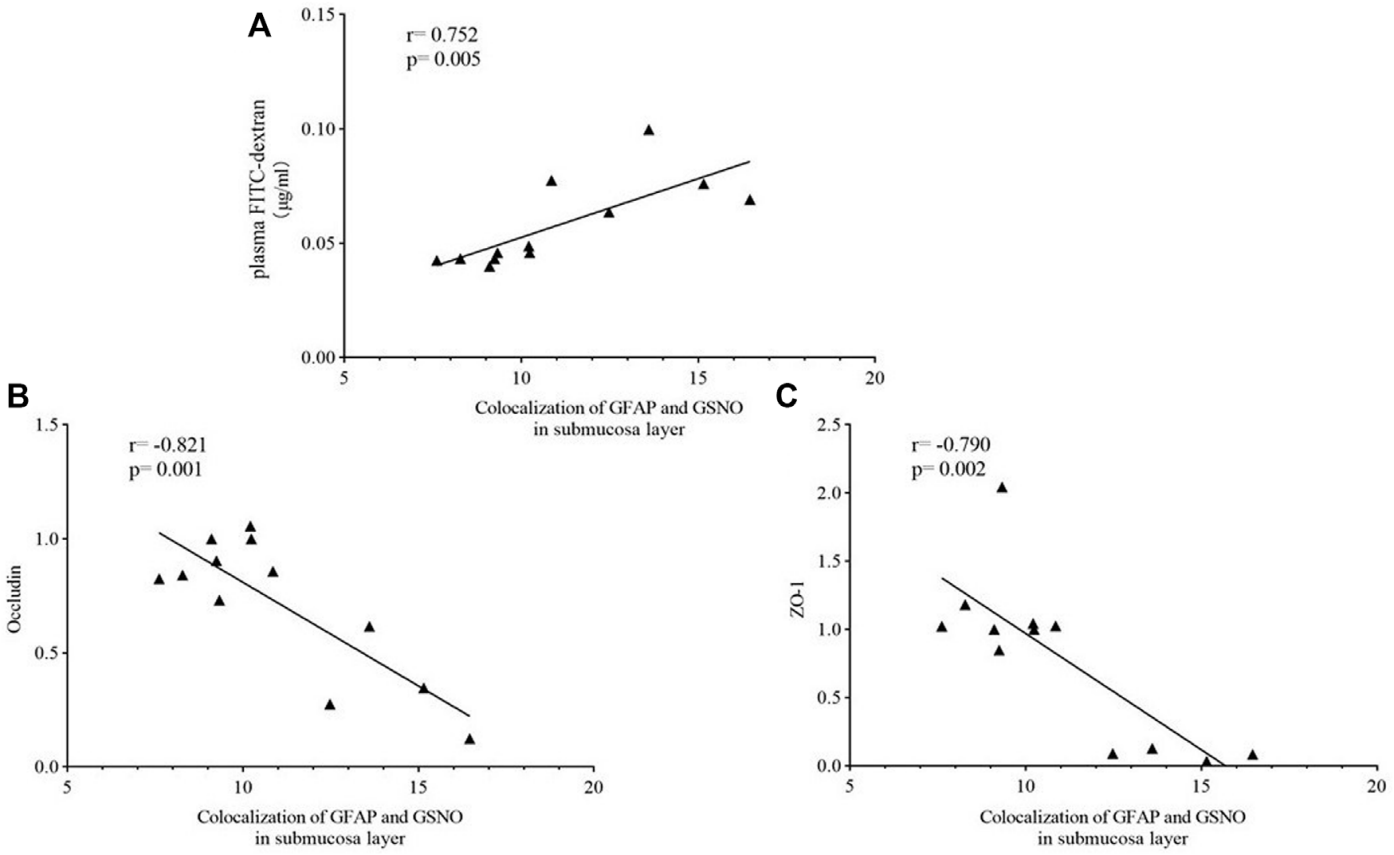
Correlation analysis between the level of EGC-derived GSNO in the submucosal layer and intestinal barrier function. Spearman test showed that significant positive correlations were observed between the expression of EGC-derived GSNO and of plasma FITC-dextran concentrations in the muscle layer **(A)**. an inverse correlation was observed between the level of EGC-derived GSNO in the muscle layer and the expressions of occludin **(B)** and ZO-1 **(C)**.

A significant positive correlation was observed between the level of EGC-derived GSNO in the submucosal layer and concentrations of plasma FITC-dextran (r = −0.752, *p* = 0.005) (Figure 12A). The level of EGC-derived GSNO in the submucosal layer was significantly inversely related to occludin (r = −0.821, *p* = 0.001) and ZO-1 (r =−0.790, *p* = 0.002) expressions (Figure 12B and Figure 12C).

## 4 Discussion

The intestinal barrier contributes to the maintenance of intestinal homeostasis (Camilleri, 2019). Once the intestinal barrier is disrupted, harmful substances such as gut microbiota and microbial metabolites cause disordered colonic luminal environment, further activate nociceptive dorsal root ganglion neurons (Groschwitz and Hogan, 2009), induce visceral hypersensitivity, and aggravate abdominal pain and diarrhea. Studies have suggested the efficacy of acupuncture against IBS from different aspects (Sun et al., 2015; Pei et al., 2018; Chen et al., 2019). The effect of acupuncture on the intestinal barrier seems notable because acupuncture repairs intestinal barrier structures, regulates tight junctions, and protects the mucus layer. Although the therapeutic effect of acupuncture has been described in IBD and other diseases, it has not been deeply studied in IBS-D. The present study suggested that EA may play a positive role in inhibiting EGC hyperactivation, regulating the GSNO expression, maintaining intestinal homeostasis by repairing the intestinal barrier, and eventually relieving IBS-D symptoms, including visceral hypersensitivity, diarrhea, and anxiety.

According to previous behavioral studies, the present study used AWR scores, DI, OT%, and histological assessment to evaluate the IBS-D model. The present study exhibited a significant increase in AWR scores, a significant decrease in OT% and DI, and no significant pathological changes in the model group. Thus, the IBS-D model was successfully established. These symptoms were significantly improved after EA treatment. This finding is concurrent with that of other studies (Chen et al., 2019; Zhao et al., 2022).

As the critical part of ENS, EGC plays a role in intestinal homeostasis maintenance. Combined with our previous studies*,* we chose EGC as a cut-off point. In addition, EGC can be observed in both the submucosal layer and muscle layer, and its function varies depending on its location (Wang et al., 2018). In the present study, TEM was performed to observe EGC. The model group exhibited autophagy and mitochondrial swelling in the submucosal layer, indicating EGC hyperactivation in the submucosal layer.

Nitric oxide (NO) is a gaseous mediator, which is involved in various pathophysiological processes, including the maintenance of intestinal homeostasis, because of its oxidative activity (Bonetti et al., 2018). Overproduction of NO may contribute to intestinal barrier dysfunction in LPS-challenged (Unno et al., 1997) and alcohol-induced liver injury rats. It is also reported that NO takes part in pain transmission and intestinal barrier regulation in IBS patients and rats (Dolatabadi et al., 2018). Among all EGC secretions, GSNO, a recently discovered physiological NO modulator molecule (Broniowska et al., 2013), has been reported to regulate blood–brain barrier and intestinal barrier function through tight junction proteins and cell adhesion molecules (Aggarwal et al., 2018). Low GSNO concentrations may act as an NO stabilizer to protect intestinal barrier function. This effect of GSNO was dose-dependent (Savidge et al., 2007). When GSNO reached a certain concentration, the opposite effects would occur (Savidge et al., 2007). The present study exhibited a significant increase in GSNO expression in the model group, suggesting that GSNO is most likely related to the intestinal barrier dysfunction in IBS-D.

The EA effect on EGC-derived GSNO was explored by observing the GFAP and GSNO colocalization in both muscle and submucosal layers. GFAP is a marker of EGC activation, and its expression exhibited a significant positive correlation with the severity of symptoms of IBS-D rats (Wang et al., 2016). A significant increase in GFAP and GSNO colocalization was observed in the model group. EA could inhibit the colocalization of GFAP and GSNO, especially in the submucosal layer. Chemical and mechanical stimuli can activate the release of mediators that would stimulate intrinsic primary afferent neurons in the submucosal plexus (da Silva et al., 2017), which may exacerbate the IBS symptoms (Yang et al., 2009). In addition, EGC in the submucosa contributes to tight junctions (Yu and Li, 2014). Thus, EGC and their secretions in the submucosal layer are highly likely to be responsible for the development of IBS.

As a crucial part of the intestinal barrier, tight junctions are vital for maintaining intestinal barrier function. Abnormalities in tight junctions disturb this sensitive balance of intestinal barrier, leading to the worsening of IBS symptoms. Studies have exhibited an obvious damage of tight junctions in patients with IBS-D, causing the increase in intestinal permeability (Hanning et al., 2021). ZO-1 and occludin, the most crucial tight junction proteins (Camilleri et al., 2012), interact directly with actin (Camilleri et al., 2012), and their expressions are lower in IBS patients (Bertiaux-Vandaële et al., 2011) as well as mice (Hou et al., 2019). FITC-dextran, a fluorescent tracer, is often used to evaluate intestinal permeability, especially in animal experience. Thus, occludin and ZO-1 expressions and plasma FITC-dextran concentrations were used to demonstrate the EA effect on intestinal barrier function. A significant increase in intestinal permeability and a significant decrease in occludin and ZO-1 expressions were observed in the model group, and EA treatment reversed the situation. Then, Spearman correlation analysis was used to evaluate the correlation between EGC-derived GSNO and intestinal barrier function. GFAP and GSNO colocalization was positively correlated with intestinal permeability and negatively correlated with the expressions of tight junction proteins. This suggests a potential role of submucosal EGC–derived GSNO in the maintenance of intestinal homeostasis in IBS-D.

Thus, submucosal EGC–derived GSNO may contribute to intestinal barrier dysfunction in IBS-D by downregulating occludin and ZO-1 expressions and cause worsening of symptoms, including visceral hypersensitivity, anxiety, and diarrhea. EA can upregulate occludin and ZO-1 expressions through submucosal EGC–derived GSNO, can restore intestinal homeostasis, and can eventually significantly improve these symptoms.

The present study has certain limitations. First, the sample size was limited, which can be enlarged in our further study. Then, considering the epidemiological characteristics that IBS-D exhibits a female predisposition, we chose female rats in this work, but it remains unknown whether sex affects EA effects, which must be explored further. In addition, an EGC inhibitor and gene knockout animals can be used and *in vitro* experiments can be performed to verify our findings at the cellular level. Further studies must be conducted to strengthen the findings of this study. The clinical efficacy of acupuncture in the treatment of IBS was relatively reliable. However, the mutual relationships among various mechanisms such as abnormal gastrointestinal motility, visceral hypersensitivity, and intestinal homeostasis disorders need to be explored.

## 5 Conclusion

The present study provides an entry point for the research on intestinal homeostasis in IBS-D, demonstrating that EA may repair intestinal barrier function in IBS-D rats possibly by upregulating occludin and ZO-1 expressions through submucosal EGC–derived GSNO.

## Data Availability

The raw data supporting the conclusion of this article will be made available by the authors, without undue reservation.
